# Chemical space as a unifying theme for chemistry

**DOI:** 10.1186/s13321-025-00954-0

**Published:** 2025-01-16

**Authors:** Jean-Louis Reymond

**Affiliations:** Department of Chemistry, Biochemistry and Pharmaceutical Sciences, University of Bern, Freiestrasse 3, 3012 Bern, Switzerland

## Abstract

Chemistry has diversified from a basic understanding of the elements to studying millions of highly diverse molecules and materials, which together are conceptualized as the chemical space. A map of this chemical space where distances represent similarities between compounds can represent the mutual relationships between different subfields of chemistry and help the discipline to be viewed and understood globally.

Aiming to understand our world, natural sciences constantly expand at the endless frontier of knowledge and become increasingly diverse. For chemistry and against Occam’s razor, matter is not simply earth, water, air and fire, let alone the hundred or so elements of the periodic table, nor is carbon the essence of the *vis vitalis*. Our field has developed a broad array of experimental methods leading to the discovery and understanding of a very large number of compositional matters ranging from materials and polymers to biomolecules and drugs, accompanied by the creation of many subfields and their specific languages [1].

Cheminformatics arose from the need to enable access to and exploitation of the chemical knowledge accumulating in the scientific and patent literature. Tools were invented to create identifiers for chemical compounds for the purpose of classification and to describe chemical structures in data formats suitable to train statistical models rationalizing the properties of known compounds and possibly predicting new ones [2, 3]. However, cheminformatics remained for many years a hidden tool supporting commercial databases, and most chemists were unaware of its potential value to guide experiments. Considering that chance favors the prepared mind, combinatorial chemistry was invented with the idea that trial and error should succeed even for difficult cases given enough trials [4]. Methods were developed to synthesize and test as many compounds as possible focusing on numbers and miniaturization [5–9]. This high-throughput screening approach for discovery, although only partly successful, popularized the evidence that discoveries in chemistry can benefit from exploiting very large datasets. In the area of medicinal chemistry, this triggered insights such as Lipinski’s rule of five [10], the assembly of open access repositories for compounds [11], and the development of molecule collections for screening [12, 13].

Screening collections were obviously commented as being “astronomically” large, suggesting using the words “chemical space” to describe the ensemble of all chemical matter, known or unknown [14–17]. Thanks to the methods developed in cheminformatics, one can formulate chemical space as a mathematical and usually high-dimensional space where distances represent similarities between molecules or materials [18, 19], and which can be represented in the form of chemical space maps by applying various dimensionality reduction methods [20–26]. In this manner, collections of molecules or materials are conceptualized as lands of opportunities to be explored by informed searches, rather than as haystacks in which to blindly search for needles. Such informed searches can greatly improve the efficiency of new discoveries in various chemistry fields such as drug discovery [27–29], chemical synthesis [30, 31], asymmetric catalysis [32, 33], materials [34–39], quantum property predictions [40], or toxicology [41].

When looking across the chemical sciences, the idea of chemical space has recently gained popularity in a very simple sense of using “a chemical space” to refer to a precise subfield of investigation such as a compound series, while ignoring the rest, which is a bit unfortunate. I would argue here that “chemical space” as a concept has the potential to do much better, specifically to unify all chemical sciences under a common roof. This would facilitate communication and the identification of cross-disciplinary opportunities and help chemistry to be viewed and understood globally. To achieve this goal will require to draft a map of chemical space representing all subfields of chemistry and their mutual relationships, not an easy task for which multiple approaches to molecular representation including artificial intelligence might be required [42–48].

## Data Availability

No datasets were generated or analysed during the current study.
